# Plasma Acylated Ghrelin Response to One Session Circuit Resistance Exercise in Fasted and High Carbohydrate Meal in Healthy Young Men

**DOI:** 10.5812/ijem.8568

**Published:** 2013-10-21

**Authors:** Marzyeh Saghebjoo, Mehdi Hedayati, Yadgar Fahimi, Saeed Ilbeigi

**Affiliations:** 1 Faculty of Physical Education and Sport Sciences, University of Birjand, Birjand, IR Iran; 2 Cellular and Molecular Research Center, Research Institute for Endocrine Sciences, Shahid Beheshti University of Medical Sciences, Tehran, IR Iran

**Keywords:** Ghrelin, Carbohydrate, Resistance Training

## Abstract

**Background:**

Ghrelin, a 28 amino acid peptide, is effective in control of appetite and body weight. Acylated ghrelin peptide is the active form of this peptide which plays a major role in the body’s energy balance.

**Objectives:**

This study aimed to investigate the possible effect (s) of intensive resistance exercise on acylated ghrelin, growth hormone, glucose, insulin, and cortisol plasma levels.

**Patients and Methods:**

Forty male students with the mean age of 19.22 ± 0.26 years and BMI 21.02 ± 0.33 (kg/m^2^) were randomly divided into experimental and control groups. Experimental group performed a single session of circuit resistance exercise with 80% 1RM in both fasting and high carbohydrate meal. Blood samples were collected before and immediately after exercise to measure the concentrations of mentioned variables.

**Results:**

Two-way ANOVA showed that acylated ghrelin and fasting plasma glucose levels after exercise in both high-carbohydrate and fasting groups were significantly increased compared to the control group (P < 0.05), but the levels of insulin, cortisol, and growth hormones did not have any significant change.

**Conclusions:**

Totally, it seems that the increased plasma acylated ghrelin during exercise is due to the decrease of muscle and liver energy stores which provides conditions for increased energy intake and positive energy balance.

## 1. Background

Some brain centers such as the hypothalamus act as central regulators of food intake, satiety, and energy homeostasis; however, hormones that are produced in adipose tissue and stomach influence these centers, leading to stimulation of appetite and feelings of fullness (1-4). In general, these hormones are involved in metabolism -that changes in their levels- affect weight and appetite (5).

Ghrelin is a 28 amino acid peptide which was first discovered in 1999 by Kojima (1). This peptide stimulates appetite and growth hormone secretion from the pituitary gland (6). The main source of this appetizing peptide is stomach which more than 70% of ghrelin in circulation is provided in this way. However, other tissues such as pancreas, placenta, kidney, pituitary, and gut are also able to secrete this hormone (7, 8). Plasma ghrelin level is regulated by nutritional and metabolic factors. In fact, ghrelin increases in negative energy balance such as before eating, malnutrition, anorexia, and fasting. Furthermore it reduces in positive energy balance, such as after eating (satiety) and also in obesity (9, 10). Plasma ghrelin is in two main forms: acylated ghrelin (AG) or active ghrelin, and unacylated ghrelin (UAG) or inactive ghrelin which constitutes 70 - 80% of the total ghrelin (11, 12). Studies have shown that ghrelin secretion depends on various factors such as nutrition (12-14), metabolism, and physical activity (15). Effect of physical activity on ghrelin levels is interesting for several reasons. Physical activity has been known as a potent stimulator of growth hormone (GH), also ghrelin stimulates GH secretion potentially. Then ghrelin can affect GH response to physical activity (15). Some studies have also demonstrated that exercise decreases ghrelin levels and hunger, which helps to reduce appetite after physical activity (16, 17). There are conflicting reports about the effects of exercise on acylated ghrelin levels. Furthermore, evaluating the effects of resistance exercise with or without carbohydrate and protein meals on appetite hormones change needs more researches.

## 2. Objectives

Since there are limited studies about the composition of diet and physical activity on ghrelin response, the present study was designed to answer this question whether a single session of circuit exercise with 80% one repetition maximum (RM) (maximum weight that a muscle or muscles group, can move only once) in fasting and high carbohydrate meal has any effect on plasma acylated ghrelin level in healthy young men.

## 3. Patients and Methods

The present study was performed using a quasi-experimental study with four groups (two control, and two experimental groups). The participants in this study were 40 active young men (Mean ± SD: age: 22.10 ± 1.08 years, height: 173.55 ± 5.03 cm, weight: 65.24 ± 7.63 kg, and body mass index: 21.02 ± 0.33 kg/m^2^) who were not involved in any weight training program during at least 3 months ago, which were randomly divided into two experimental and two control groups.

Inclusion criteria were absence of history of cardiovascular, respiratory, and renal failure diseases. Subjects were also not treated with steroid drugs and special diets (low calorie, low fat, and high protein). Before training, subjects’ demographic characteristics such as age, weight, height, body fat percentage, and BMI were measured. The amount of one repetition maximum of nine exercises used in the resistance experimental group was calculated using the following formula (18):

Predicted 1 - RM = weight lifted/ 1.0278 – 0.278 X

Where X = The number of reps performed

Training program was designed as a circuit program using free weights and equipment. Exercises included bench press, leg press, seated rowing, overhead press, leg extension, triceps extension, leg curl, arm curl, and heel raise. The exercise session consisted of three circles in which nine of those were performed in tandem. Each movement was performed for 30 seconds (8 times with 80% one RM), resting 30 seconds between movements, and resting between the two circles were 120 seconds. The total time for each session was 55 to 50 minutes, including:

- Warm up for 15 to 20 minutes, it was very light and without resistance exercise,

- Weight training for 30 minutes,

- Cool down for 5 minutes (1),

Control and experimental groups were asked to avoid usual food intake until the night before sampling. The breakfast package included 120 g bread, 20 g honey, and 1 cup sweetie tea which was considered as a high carbohydrate diet based on nutritional aspect. A carbohydrate breakfast package was prepared for each subject for high-carbohydrate diet. To prepare carbohydrate breakfast the resting metabolic rate (RMR) and total calories for each person per day were calculated using the Harris-Benedict method as follows (19):

RMR = 66.473 + 13.751 (weight) + 5.0033 (height) - 6.751 (age)

Total energy consumption = 1.66 × RMR

Breakfast calories = Total energy consumption × 20%

Then 20% of total calculated calorie was considered as breakfast meal (20).

Control and experimental groups were also asked to maintain their dietary restrictions from 8PM, the night before sampling, and to eat their high carbohydrate content breakfast at 5 - 6 AM. Then 10ml blood was taken from the brachial vein at 8 AM. Sampling of all the test subjects of control and experimental groups was performed immediately after the training session. Blood samples were collected in tubes containing anticoagulant (EDTA), and were immediately centrifuged (2000 rpm, 10 minutes); obtained plasma was divided into two separate tubes which were maintained at -80°C to be used for the measurement of acylated ghrelin, insulin, growth hormone, cortisol, and plasma glucose. Acylated ghrelin, insulin, cortisol, and glucose levels in plasma samples were measured by sandwich ELISA method. The sensitivity of these methods was 3.9 pg/mL, 1 mg/L, 0.4 µg/dL, 0.2 ng/mL for acylated ghrelin, insulin, cortisol, and glucose, respectively. Also the intra coefficient of variation was 6.2%, 4.5%, 6.1%, and 2.7%, respectively. Plasma glucose was measured by enzymatic colorimetric method using glucose oxidase kit (Pars Azmun, Iran). The sensitivity of this method was 5 mg/dL, and its intra coefficient of variation was 1.7%.

The Kolmogorov-Smirnov test was used to check the normality of data distribution. To compare the mean variation before and after the test, parametric two-way analysis of variance (Two-way ANOVA) and paired t-test were used. Statistical analysis was performed using SPSS version 15. The level of significance was considered P < 0.05.

## 4. Results

Table 1 shows the demographic characteristics of the participants. Table 2 summarizes the results from two-way ANOVA. This results show significant increase of plasma acylated ghrelin levels in the experimental group after exercise (P = 0.037). Also nutrition (P = 0.23) and exercise-nutrition interaction (P = 0.11) did not lead to any significant differences in plasma acylated ghrelin levels. Despite post-test plasma acylated ghrelin levels were not significantly different in the two experimental groups; paired t-test was used to analyze changes in these variables at pretest to post-test in the experimental groups. The findings of this test showed that acylated ghrelin plasma levels after exercise were significantly increased in the fasting state (P = 0.03, 81%), while this variable did not significantly increase after exercise in carbohydrate-rich diet state (P = 0.54, 11%). Plasma glucose levels in the experimental group showed a significant increase in comparison with the control group (P = 0.049), while nutrition (P = 0.16) and exercise-nutrition interaction (P = 0.46) did not have significant change on plasma glucose levels. Changes in levels of growth hormone, plasma insulin, and cortisol following circular resistance training in both fasting and fed high-carbohydrate were not significant (P values, 0.11, 0.68, and 0.68 respectively) (Table 2). 

**Table 1.** Individual Characteristics of Subjects in Experimental and Control Groups (Mean ± SD)

Variables	Groups			
	Fast Control, n = 9	Fed Control, n = 10	Fast Exercise, n = 10	Fed Exercise, n = 11
**Age, y**	22.33 ± 0.86	22.10 ± 1.08	22.00 ± 1.32	21.90 ± 1.28
**Height, cm**	176.11 ± 3.98	174.70 ± 2.86	171.55 ± 5.34	172.63 ± 6.23
**Weight, kg**	67.84 ± 9.18	69.98 ± 6.47	61.47 ± 5.51	62.23 ± 6.47
**BMI, kg/m2**	21.55 ± 2.05	20.88 ± 1.43	21.33 ± 1.74	21.16 ± 1.76
**Percentage of body fat**	14.73 ± 4.56	14.92 ± 2.64	14.01 ± 3.40	13.41 ± 3.22

**Table 2.** Plasma Levels of Acylated Ghrelin, Insulin, Cortisol, Growth Hormone, and Glucose in Subjects (Mean ± SD)

Variables	Time	Groups				P Values		
		Fast Control, n = 9	Fed Control, n = 10	Fast Exercise, n = 10	Fed Exercise, n = 11	Exercise	Nutrition	Exercise-Nutrition Interaction
**Acylated ghrelin, pg/mL**	Pre-test	18.17 ± 19.02	25±15.06	22.30 ± 16.53	22.30 ± 11.84	0.03^a^	0.23	0.11
	Post-test	18.44 ± 12.14	22.73±18.03	40.50 ± 37.18	25.60 ± 15.83			
**Insulin, mU/L**	Pre-test	2.9 ± 1.19	14.57±5.97	3.70 ± 1.44	15.44 ± 5.34	0.68	0.01^a^	0.28
	Post-test	3.01 ± 1.65	7.25±4.69	5.66 ± 2.18	7.30 ± 5.71			
**Cortisol, µg/dL**	Pre-test	27.70 ± 3.42	28.33 ± 5.07	29.77 ± 4.01	27.87 ± 8.41	0.68	0.88	0.49
	Post-test	35.50 ± 9.04	23.96 ± 4.62	36.80 ± 10.77	36.28 ± 12.58			
**Growth hormone, ng/mL**	Pre-test	0.41 ± 0.53	0.42 ± 0.55	1.70 ± 2.67	0.25 ± 0.03	0.11	0.84	0.21
	Post-test	1.18 ± 1.34	0.27 ± 0.07	2.71 ± 1.73	1.93 ± 1.41			
**Glucose, mg/dL**	Pre-test	76.66 ± 7.43	81.70 ± 16.24	81.70 ± 11.55	79.80 ± 14.19	0.04^a^	0.16	0.46
	Post-test	18.17 ± 19.02	77.9 ± 10.96	93.60 ± 9.39	88.63 ± 13.09			

^a^P < 0.05

## 5. Discussion

The findings of this study showed that a single session of circular resistance exercise leads to significant increased levels of acylated ghrelin and plasma glucose in both fasting and high carbohydrate feeding groups. Also plasma levels of insulin, cortisol, and growth hormones were not affected by exercise and nutrition-exercise interaction.

Some studies have shown that acylated ghrelin is sensitive and changeable with weight loss and negative energy balance (7, 21). Sauseng and colleagues reported a significant increase of plasma acylated ghrelin after short-term controlled exercise in school children, while total ghrelin remained unchanged. Increased acylated ghrelin following exercise could be due to the absorption of calorie to offset energy costs, which is a physiological response (21). Hosoda and colleagues reported that acylated ghrelin can respond more quickly to glucose rather than total ghrelin, this event could be due to the glucose consumption by cells and intensive activity; it means that the change of acylated ghrelin percentage in response to glucose is greater than total ghrelin (7). Several studies have demonstrated that weight training increases glycogen breakdown and leads to energy deficit (22). After intense exercise, protein and glycogen synthesis reduces, which are factors to secrete ghrelin, and increased energy intake (23). In the present study, no significant differences were observed among pretest acylated ghrelin levels in fasting and fed groups. In several studies about the impact of food on ghrelin secretion, most researchers have concluded that ghrelin secretion is reduced subsequent of food intake (24, 25), they demonstrated that the velocity of calorie intake and energy absorption is very important for ghrelin levels reduction. In the study of Francesco et al. following carbohydrate intake in older subjects, the acylated ghrelin curve reduced, hence researchers proposed a significant role of age in the acylated ghrelin suppression (23). Also in the study of Blom et al. following food intake of carbohydrate and protein, ghrelin levels were significantly reduced (26). It seems that one of the most important reasons for the differences in ghrelin levels in fasted and fed groups at pretest of present study is carbohydrate-rich breakfast consumption about three hours before sampling. Therefore the blood glucose level during this time has returned to its normal level, and its inhibitory effect on acylated ghrelin was also deleted.

The result of this study about increased acylated ghrelin levels after exercise is consistent with the studies that have expressed ghrelin levels increase after exercise due to the energy recovery and rebuilding lost glycogen stores during exercise, which increases appetite, provides food and positive energy balance. In this study, the amount of fasting acylated ghrelin levels in post-test groups was higher than the fed experimental group. In this study it seems that this result is due to a decrease of muscle carbohydrate stores after exercise in fasting group compared to the high-carbohydrate group. Other findings from the study showed a significant increase in both fasting plasma glucose and high carbohydrate meal immediately after exercise.

In our study high intensity exercise appeared to stimulate the release of catecholamines and also facilitated hepatic glycogenolysis which results in a significant increase of plasma glucose. But since the present study did not measure the levels of catecholamines, it is necessary to be more cautious about this issue. Following exercise, plasma glucose levels are decreased due to the entry of glucose into muscle to replace depleted muscle glycogen stores with glucose (27). On the other hand, some studies have described that increased plasma glucose during exercise can be caused by changes in plasma volume (21). Since in our study changes in plasma volume has not been calculated, probably the reduction in plasma volume due to the high intensity activity is one the possible reasons for the increase of plasma glucose levels immediately after exercise.

So far, there is no obvious reason for the correlation between insulin and ghrelin hormones. It seems that hyperinsulinemia is a feedback mechanism to inhibit ghrelin secretion (24). Based on the present results the amounts of these hormones were not consistent with each other after exercise. Probably one of the reasons for the lack of change in insulin in this study is the use of high intensity exercise which can lead to the accumulation of lactic acid in the body. In addition increased blood lactate and pH are inhibitory factors on insulin secretion. In a study plasma ghrelin, cortisol, and glucose levels increased in response to the intense exercise, but insulin level remained unchanged. One reason for the lack of significant changes in cortisol in this study can be attributed to the results of few and colleagues in which the intense exercise increased the uptake of cortisol by peripheral tissues, following the increase intensity, retention of cortisol from tissue to plasma is observed (28).

In the present study, growth hormone changes following resistance activity is consistent with the circular acylated ghrelin changes. Initial experiments in rodents and clinical studies have shown that ghrelin is a growth hormone-releasing factor. However, no significant association was observed between plasma ghrelin levels and GH levels, yet. The results show that both ghrelin and growth hormones increase during starvation. In fact, ghrelin may stimulate an unidentified hypothalamic agent (U-factor) which, in turn, stimulates GH release (29). So these reasons could justify the results of our study.

Totally, in the present study ghrelin levels after exercise and energy expenditure were increased to compensate for the energy lost and to rebuild muscle glycogen stores which causes increasing appetite and positive energy balance. The important point in this study is that the amount of acylated ghrelin increase in posttest of fasting exercise group was significantly more than the non-fasting exercise group. Presumably, glucose store in muscle cells of fasting group was much more robust, which is an intense stimulus for increased expression of acylated ghrelin.

It appears that plasma acylated ghrelin changes is very sensitive to changes in conditions of negative energy balance and its increase is an effort to compensate for the increased appetite and lost energy reserves.

## References

[1] Kojima M, Hosoda H, Date Y, Nakazato M, Matsuo H, Kangawa K (1999). Ghrelin is a growth-hormone-releasing acylated peptide from stomach.. Nature..

[2] Saghebjoo M, Ghanbari-Niaki A, Rajabi H, Fathi R, Hedayati M (2011). [Effects of circuit resistance training on plasma ghrelin levels in young women. Iranian Journal of Endorinology and Metabolism]..

[3] Wynne K, Stanley S, McGowan B, Bloom S (2005). Appetite control.. J Endocrinol..

[4] Speakman JR (2004). Obesity: the integrated roles of environment and genetics.. J Nutr..

[5] Hedayati M, Saghebjoo M, Ghanbari-Niaki A (2012). Effects of circuit resistance training intensity on the plasma ghrelin to obestatin ratios in healthy young women.. Int J Endocrinol Metab..

[6] Kojima M, Hosoda H, Matsuo H, Kangawa K (2001). Ghrelin: discovery of the natural endogenous ligand for the growth hormone secretagogue receptor.. Trends Endocrinol Metab..

[7] Hosoda H, Kojima M, Mizushima T, Shimizu S, Kangawa K (2003). Structural divergence of human ghrelin. Identification of multiple ghrelin-derived molecules produced by post-translational processing.. J Biol Chem..

[8] Bang AS, Soule SG, Yandle TG, Richards AM, Pemberton CJ (2007). Characterisation of proghrelin peptides in mammalian tissue and plasma.. J Endocrinol..

[9] Moran LJ, Luscombe-Marsh ND, Noakes M, Wittert GA, Keogh JB, Clifton PM (2005). The satiating effect of dietary protein is unrelated to postprandial ghrelin secretion.. J Clin Endocrinol Metab..

[10] Mirzaei B, Irandoust K, Rahmani-Nia F, Mohebbi H, Hassan-Nia S (2009). Unacylated ghrelin levels increase after aerobic exercise in obese women.. Brazilian J Biomotric..

[11] Zwirska-Korczala K, Konturek SJ, Sodowski M, Wylezol M, Kuka D, Sowa P (2007). Basal and postprandial plasma levels of PYY, ghrelin, cholecystokinin, gastrin and insulin in women with moderate and morbid obesity and metabolic syndrome.. J Physiol Pharmacol..

[12] Soriano-Guillen L, Barrios V, Martos G, Chowen JA, Campos-Barros A, Argente J (2004). Effect of oral glucose administration on ghrelin levels in obese children.. Eur J Endocrinol..

[13] Tschop M, Smiley DL, Heiman ML (2000). Ghrelin induces adiposity in rodents.. Nature..

[14] Tannous dit El Khoury D, Obeid O, Azar ST, Hwalla N (2006). Variations in postprandial ghrelin status following ingestion of high-carbohydrate, high-fat, and high-protein meals in males.. Ann Nutr Metab..

[15] Kraemer RR, Castracane VD (2007). Exercise and humoral mediators of peripheral energy balance: ghrelin and adiponectin.. Exp Biol Med (Maywood)..

[16] Hamedinia M, Davarzani Z, Hosseini kakhk A (2011). [The effect of one session of swimming and running training on hunger rate and ghrelin, insulin and cortisol hormones of the plasma in the healthy girls].. Iran J Endorinol and Metab..

[17] Ballard TP, Melby CL, Camus H, Cianciulli M, Pitts J, Schmidt S (2009). Effect of resistance exercise, with or without carbohydrate supplementation, on plasma ghrelin concentrations and postexercise hunger and food intake.. Metabolism..

[18] Brzycki M (1993). Strength testing—predicting a one-rep max from reps-to-fatigue.. JOPERD..

[19] Harris JA, Benedict FG (1919). A Biometric Study of Basal Metabolism in Man..

[20] Foster-Schubert KE, Overduin J, Prudom CE, Liu J, Callahan HS, Gaylinn BD (2008). Acyl and total ghrelin are suppressed strongly by ingested proteins, weakly by lipids, and biphasically by carbohydrates.. J Clin Endocrinol Metab..

[21] Sauseng W, Nagel B, Gamillscheg A, Aigner R, Borkenstein M, Zotter H (2011). Acylated ghrelin increases after controlled short-time exercise in school-aged children.. Scand J Med Sci Sports..

[22] Ghanbari-Niaki A (2006). Ghrelin and glucoregulatory hormone responses to a single circuit resistance exercise in male college students.. Clin Biochem..

[23] Di Francesco V, Fantin F, Residori L, Bissoli L, Micciolo R, Zivelonghi A (2008). Effect of age on the dynamics of acylated ghrelin in fasting conditions and in response to a meal.. J Am Geriatr Soc..

[24] Volek JS (2004). Influence of nutrition on responses to resistance training.. Med Sci Sports Exerc..

[25] Tesauro M, Schinzari F, Caramanti M, Lauro R, Cardillo C (2010). Metabolic and cardiovascular effects of ghrelin.. Int J Pept..

[26] Blom WA, Lluch A, Stafleu A, Vinoy S, Holst JJ, Schaafsma G (2006). Effect of a high-protein breakfast on the postprandial ghrelin response.. Am J Clin Nutr..

[27] Wilmore JH, Costill DL (1994). Physiology of sports and exercise..

[28] Few JD (1974). Effect of exercise on the secretion and metabolism of cortisol in man.. J Endocrinol..

[29] Horvath TL, Diano S, Sotonyi P, Heiman M, Tschop M (2001). Minireview: ghrelin and the regulation of energy balance--a hypothalamic perspective.. Endocrinology..

